# Transcriptomics in Alzheimer’s Disease: Aspects and Challenges

**DOI:** 10.3390/ijms21103517

**Published:** 2020-05-15

**Authors:** Eva Bagyinszky, Vo Van Giau, SeongSoo A. An

**Affiliations:** 1 Department of Industrial and Environmental Engineering, Graduate School of Environment, Gachon University, Seongnam 13120, Korea; navigator120@gmail.com; 2 Department of Bionano Technology, Gachon University, Seongnam 13120, Korea

**Keywords:** trancriptome, differently expressed genes, Alzheimer’s disease, neurodegeneration, noncoding RNA, alternative splicing, copy number variant, RNA array, RNA sequencing

## Abstract

Alzheimer’s disease (AD) is the most common cause of dementia. Although the heritability of AD is high, the knowledge of the disease-associated genes, their expression, and their disease-related pathways remain limited. Hence, finding the association between gene dysfunctions and pathological mechanisms, such as neuronal transports, APP processing, calcium homeostasis, and impairment in mitochondria, should be crucial. Emerging studies have revealed that changes in gene expression and gene regulation may have a strong impact on neurodegeneration. The mRNA–transcription factor interactions, non-coding RNAs, alternative splicing, or copy number variants could also play a role in disease onset. These facts suggest that understanding the impact of transcriptomes in AD may improve the disease diagnosis and also the therapies. In this review, we highlight recent transcriptome investigations in multifactorial AD, with emphasis on the insights emerging at their interface.

## 1. Introduction

Alzheimer’s disease is a complex disease since several genetic and epigenetic factors and gene–environmental interactions could be involved in disease onset. Neuropathological changes in the AD brain include progressive hippocampal and cortical atrophy, visible upon neuroimaging and macroscopic examination, suggesting intracellular neurofibrillary tangles (NFTs) of the hyperphosphorylated tau protein and extracellular depositions of amyloid-β (Aβ)1–42 peptide accompanied by neuronal and synapse loss and reactive gliosis [1]. Molecular genetic investigation of these pedigrees have resulted in the identification of the amyloid precursor protein (APP), presenilin 1 (PSEN1), and presenilin 2 (PSEN2), and several risk factors were identified, which may impact AD onset. *APP, PSEN1*, and *PSEN2* could be causative factors for AD with an earlier age of onset (early onset AD, EOAD, under 65 years). The majority of mutations in these genes could be associated with autosomal dominant inheritance. However, these mutations may be rare even among the early-onset AD (EOAD) patients. Several rare risk variants in Sortilin Related Receptor 1 (*SORL1*), triggering receptors expressed on myeloid cells (*TREM2*), andATP Binding Cassette Subfamily A Member 7 (*ABCA7*) may contribute to EOAD risk [2]. Apolipoprotein E (*APOE*) E4 allele has been identified as the main risk factor for late-onset AD (LOAD), but it may not define all disease cases [3]. *APOE* E4 explains approximately 25% of heritability in AD. In recent years, genome-wide association (GWAS), next-generation sequencing (NGS), and whole-genome/exome (WGS/WES) sequencing analyses have provided more insight into AD genetics. Several low penetrant common risk variants and rare mutations were also discovered, which could also impact the risk of AD or act as risk modifiers, such as clusterin (*CLU), SORL1, ABCA7,* Siglec-*3 (CD33*), Phospholipase D Family Member 3 (*PLD3*), Phosphatidylinositol Binding Clathrin Assembly Protein (*PICALM*), NME/NM23 Family Member 8 (*NME8), TREM2,* A-Kinase Anchoring Protein 9 (*AKAP9*), or A Disintegrin and metalloproteinase domain-containing protein 10 (*ADAM10*) [4,5,6,7,8]. A combination of common variants (which minor allele frequency is more than 5%) may have a significant impact on AD onset. Variants in these genes may not impact strongly on the AD risk by themselves, but their combination could have a stronger correlation with disease onset. Polygenic risk scores, based on common variants, may be useful in the estimation of AD risk. These genes with common risk variants could usually be involved in lipid metabolism, inflammatory pathways, or endocytosis. [9,10,11]. Although an emerging number of genes have been suggested to affect the risk of developing AD, their mechanistic insights and improved disease management remain limited due to difficulties in determining the functional consequences of genetic associations. Knowledge of the translational impact of these findings remained limited. Studies are ongoing on genetic variants in AD risk genes or candidates, and on the physiological complexity of tissues. Genome-wide association studies (GWAS), next-generation sequencing (NGS), and whole-exome/genome sequencing (WES/WGS) studies on large AD cohorts could provide a more detailed image on disease-associated factors [7,8]. Finding relationships between gene dysfunctions and different pathological mechanisms, such as neuronal transports, APP processing, calcium homeostasis, and impairment in mitochondria, should be important [12,13]. 

**Figure 1:**
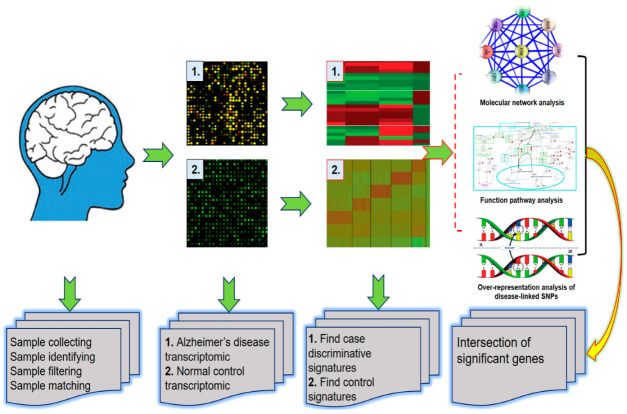
Overview showing the workflow for transcriptomic studying in Alzheimer’s disease, from transcriptomic data generation to integration of regulatory information to assess gene regulatory networks.

Emerging studies revealed that alternative gene expression regulation mechanisms, such as mRNA-transcription factor interactions, non-coding RNAs, alternative splicing, or copy number variants, could also impact neurodegeneration (Figure 1) [14]. More trends are emerging on simultaneous interrogation of transcriptome data to study the effect of newly identified genetic risk factors at the level of the transcriptome [14]. Hence, this work aims to describe and discuss the genetic discovery and transcriptome investigations in multifactorial AD, with emphasis on the insights emerging at their interface. Keywords used to search the review topics were transcriptomes, Alzheimer’s disease, neurodegeneration, differently expressed genes, non-coding RNAs, alternative splicing, copy number variant (CNV), gene expression array, RNA sequencing, and miRNA-based methods.

## 2. Differentially Expressed Genes (DEGs) in AD

Differentially expressed genes (DEGs) are important targets in the discovery of biological pathways involved in different diseases, such as cancers or neurological diseases. The goal of DEG analysis is to find genes that could be up- or downregulated in disease, compared to unaffected controls. Over- or underexpression of different genes may result in alterations in metabolic, immune, and other pathways, leading to diseases [15,16]. DEGs could also impact the onset of neurodegenerative diseases, including Alzheimer’s disease. In addition, variations may be possible between the gene expression patterns of different brain areas [17]. It is important to find out whether the transcriptional changes could result in additive effects in the known disease risk factors and disease-related pathways. The occurrence of AD may increase with age, and abnormal transcriptional changes result in disease-associated mechanisms [18,19]. 

Single-cell expression analysis revealed that several genes could be associated with neurofibrillary tangle pathology. DEGs were compared in AD-NFT neurons, AD-non-NFT neurons, and normal non-NFT neurons. Expression casein kinase 2, beta polypeptide (*CSNK2B*), apolipoprotein J (*APOJ*), and tissue inhibitor of metalloproteinase 3 (*TIMP3*) interleukin-1 receptor-associated kinase 1 (*IRAK1*) were both upregulated in both AD-NFT and AD-non-NFT neurons compared to normal neurons. In addition, their expression was higher in AD-NFT neurons, compared to AD-non-NFT neurons. Expression of calpain 7 (*CAPN7*) was repressed in AD neurons, both with NFT and non-NFT pathology [20]. Whole transcriptome analysis was performed by Antonnel et al. (2013), who compared the alternative expression of EOAD cases with- and without *PSEN1* mutations. This study did not find any significant difference between the DEG pattern of *PSEN1* positive and *PSEN1* negative EOAD cases [21]. However, several genes were differently expressed between controls and EOAD cases (with or without mutation). DEGs were involved in several mechanisms, including the calcium signaling pathway, neuroactive ligand–receptor interaction, microtubule-associated protein tau (MAPT) signaling, long term potentiation, or axon guidance [21]. Canchi et al. (2019) compared the expression pattern of 414 AD patients and unaffected individuals. They combined brain-tissue specific brain interactions with gene networks and found different gene clusters, which expression levels may change in AD [22]. These clusters include the synaptic transmission (Neuregulin 1, *NRG1*; Gamma aminobutyric acid, *GABA*; Lysophosphatidic Acid Receptor 1, *LPAR*), DNA repair and transcription (Minichromosome Maintenance Complex Component 7, *MCM7*; Tyrosyl-DNA phosphodiesterase 1, *TDP1*; DEK Proto-Oncogene, *DEK*), immune response (*TYROBP, CXCR4*, *SOCS3*), uncharacterized candidates (Rho GTP-ases, ADP ribolsylation factors), and metabolic factors (Dynamin 1 Like, *DNM1L*; HIG1 Hypoxia Inducible Domain Family Member 1A, *HIGD1A*; Cytochrome C Oxidase Assembly Factor Heme A:Farnesyltransferase, *COX10*). Gene enrichment analysis revealed that four targets were enriched and differentially expressed in all clusters, including Sp1 Transcription Factor (*SP1*), Early Growth Response 3 (*EGR3*), TGFB Induced Factor Homeobox 1 (*TGIF1*), Bromodomain PHD Finger Transcription Factor (*BPTF*). No association has been found between *EGR3* and AD yet. *EGR3* downregulation may play a role in dysfunctions of the synaptic vesicle cycle. *EGR3* could directly interact with VGF Nerve Growth Factor Inducible (VGF) and Vacuolar-type H+-ATPase (V-ATPASE), and the impairment of this pathway could impact the expression of different genes, such as Synaptosome Associated Protein 25 (*SNAP25*), Clathrin Heavy Chain (*CLTC*), resulting in reduced vesicle docking and recycling, respectively [22]. A multi-scale study by Morabito et al. (2019) across different brain datasets revealed AD specific transcriptomic signatures. Several pathways were downregulated, such as ubiquitination, mitochondrial functions, and synaptic transmissions. Immune-associated glial modules and Janus kinase (JAK)-signal transducer/activator of transcription (JAK/STAT) and mitogen-activated protein kinase (MAPK) signaling were upregulated in AD [23]. This study also found an alteration in the expression of long non-coding RNAs [23]. Whole transcriptome sequencing on the hippocampus by van Rooij et al. (2019) revealed 2716 DEGs, from which 735 were clustered into 33 modules by gene ontology biological process (GOBP) terms [24]. These modules could be involved in signal transduction, transport, or cellular metabolism. DEGs also included 2 known AD risk genes: Myocyte Enhancer Factor 2C (*MEF2C*) and Protein Tyrosine Kinase 2 Beta (*PTK2B*). However, the discovery modules may also interact with several AD risk genes, including (Major Histocompatibility Complex, Class II, DR Beta 1/5) *HLA-DRB1/HLA-DRB5*, Bridging Integrator 1 (*BIN1*), *PICALM* (endocytosis and signaling), *ABCA7* and *MAPT* (ion transport), *APP* (signal transduction), or *CLU* (exocytosis) [24]. Single-cell transcriptome analysis by Mathys et al. analyzed different cell types in the postmortem brains of AD patients, including excitatory neurons, oligodendrocytes, microglia, endothelial cells, or pericytes. This study found 1031 DEGs in nerve cells compared to the brain of patients and unaffected individuals. The majority of DEGs were related to cell-related processes. Disturbances of expression in myelination associated genes were found in all cell types, especially in oligodendrocytes and their progenitor cells. Several genes presented critical changes in gene expression, such as Leucine Rich Repeat And Ig Domain Containing 1 (*LINGO1*), Erbb2 Interacting Protein (*ERBB2IP*), Contactin Associated Protein 2 (*CNTNAP2*), Neuronal Growth Regulator 1 (*NEGR1*), Brain Expressed X-Linked 1 (*BEX1*), or Netrin G1 (*NTNG1*). In early disease stages, more cell-specific but upregulated expression changes and genes associated with stress response and maintaining homeostasis, respectively, became more common in all cell types. Differences were observed between male and female patients in expression patterns, especially in the neurons and oligodendrocytes. In males, amyloid pathology was associated with oligodendrocyte activation, while in females, downregulation was found in excitatory and inhibitory neurons [25].

## 3. Regulatory Non-Coding RNAs in AD

Non-coding RNAs (ncRNAs) are never translated into proteins, and their role in any disorder has remained unclear for a long time. However, they were verified to play a role in gene expression and gene-environment interactions as regulators. NcRNAs could affect the transcription translation by binding DNAs, coding RNAs, or proteins. Two groups of ncRNAs may be distinguished: housekeeping and regulatory ncRNAs. The housekeeping ncRNAs, such as tRNAs, rRNAs, or small nuclear RNAs (snRNAs), are expressed in all kinds of cells. They play an essential role in maintaining healthy cell functions. Regulatory ncRNAs, such as miRNA, long non-coding RNA (lncRNA), and endogenous small interfering RNAs (siRNA), are expressed in case of a certain stimulus from the environment or specific internal signal in certain cell types. [26,27]. Initially, ncRNAs were suggested to be only linked to cancers, but emerging evidence is available on their role in different brain functions, such as neural development, synaptic plasticity, transmission, or brain aging [26].

Long non-coding RNAs (LncRNAs) are longer than 200 nucleotides, have conserved structure, and are in both cytoplasm and nucleus. Their function in disease is not well understood, but they are suggested to be involved in different physiological pathways, including genomic imprinting, immune functions, or developmental processes [28]. Zhou and Xu (2015) revealed that their expression may alter in the case of AD. This study analyzed post mortem brain tissue samples and found 24 upregulated and 84 downregulated lncRNAs in patients, compared with unaffected controls [29]. Gene Set Enrichment Analysis (GSEA) revealed that the lncRNA n341006, involved in protein ubiquitination, was strongly downregulated. Expression of several genes, involved ubiquitin-associated pathways (such as Tripartite Motif Containing 23, *TRIM23*; Ubiquitin Conjugating Enzyme E2 N, *UBE2N*; Ubiquitin B, *UBB*) were correlated with n341006. This study also found significant upregulation of lncRNA n336934, which may play a role in cholesterol homeostasis [29]. LncRNAs may also impact the APP cleavage since the expression of lncRNA BACE1-AS could be upregulated in AD patients and result in enhanced beta-secretase cleavage. In vitro studies on SH-SY5Y cells revealed that silencing lncRNA Beta-Secretase 1 antisense RNA (BACE-1-AS) may downregulate the beta-secretase cleavage [30]. BC200 was suggested to be involved in the synaptic plasticity. BC200 was upregulated in the brain of AD patients, such as the hippocampus and prefrontal association cortex. Overexpression of BC200 may be associated with synaptodendritic dysfunctions, resulting in AD-associated neurodegeneration [31]. Lnc-NDM29 (neuroblastoma differentiation marker 29) has been suggested to play a role in neurodegeneration, since it may induce APP synthesis. Overexpression of Lnc-NDM29 may induce the beta- and gamma-secretase cleavage, resulting in enhanced amyloid generation. It was suggested that inflammatory stimuli could increase the expression of Lnc-NDM29, and inhibiting Lnc-NDM29 could be a potential target of anti-inflammatory drug development [32]. BC200 may be an important mediator of BACE1 activity, since blocking BC200 may reduce beta-secretase expression and amyloid peptide production [33]. Lnc- Metastasis Associated Lung Adenocarcinoma Transcript 1 (MALAT1) regulates the apoptosis and growth of neurons and inflammation. Upregulated MALAT-1 expression has been suggested to have neuroprotective and anti-inflammatory effects in AD and other neurodegenerative diseases (spinal cord injury, multiple sclerosis) [34]. Studies on AD rat models confirmed the protective effects of MALAT-1 overexpression. In addition, enhanced expression of Lnc-MALAT-1 may be correlated with the expression of miR-125 mediated genes (such as Prostaglandin-Endoperoxide Synthase 2, *PTGS2*; Cyclin Dependent Kinase 5, *CDK5*; and Forkhead Box Q1, *FOXQ1*), resulting in enhanced neurite overgrowth [35]. Lnc-51A is a non-characterized transcription unit located in the intron-1 of the *SORL1* gene. Expression of Lnc-51A was upregulated in the brain of AD patients, resulting in the increased expression of abnormally spliced *SORL1* and an elevated degree of amyloid formation [36]. Lnc-17A is located in the intronic region of the GPR51 (GABA B2 receptor) gene, and it may control its maturation. Abnormal overexpression of Lnc-17A may induce the alternative splicing of *GPR51*, and reduce the expression of full-length GABAB R2. This process could result in the inhibition of GABAB R2 signaling. Lnc-17A could stimulate the inflammatory pathways (such as IL-17A), and induce the brain inflammation. Interestingly, Lnc-17A overexpression could also enhance amyloid production through NMDA receptors [37]. 

**Figure 2:**
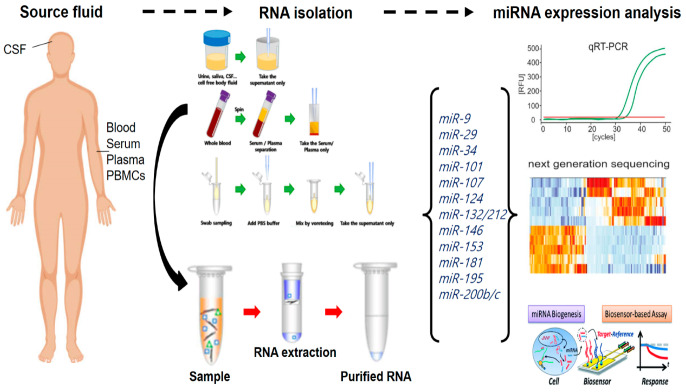
Methodological aspects to consider in most common miRNA biomarkers for Alzheimer’s research.

MicroRNAs are composed approximately 20 nucleotides and bind to the 3‘- UTR region of target mRNAs. This binding may result in blocking the translation of certain genes or in the degradation of mRNA. MiRNAs play an important role in the regulation of brain functions and neuronal development. Imbalance in miRNA expression was verified as an important factor in AD-related pathways [38]. Expression of miRNAs may be up- or downregulated in AD. MiRNAs may act through aging, and they could reduce the neuroprotection and induce inflammatory mechanisms, oxidative stress, or protein misfolding. MiRNAs may be associated with AD through APP processing, amyloid formation, and Tau phosphorylation. In addition, exosomal miRNAs within biological fluids are known as promising disease-related markers and have emerged as a powerful tool for solving many difficulties in both the diagnosis and treatment of AD patients. In AD, for example, miRNA profiling experiments (in brain tissue) have resulted in the identification of many disease-specific miRNAs that have been confirmed independently in many studies [39]. For example, hsa-miR-106, hsa-miR-153, and hsa-miR-101 have been shown to modulate APP [40,41,42], while beta secretase 1 (BACE1) has been shown to be targeted by hsa-miRNA-29 and hsa-miR-107, linking miRNAs to the regulation of amyloid production in AD brains [43]. Based on similar studies, researchers have focused on these disease-specific miRNAs to determine if differential levels are found in more-easily accessible biofluids like blood or CSF. Table 1 introduces several miRNAs involved in different AD-associated mechanisms, including amyloid metabolism, Tau pathology, immune mechanism, or cell death. MiRNAs in biofluids (blood, plasma, serum, saliva) were believed to be promising markers for AD diagnosis [44]. For better understanding of the disease mechanisms and for early detection and diagnosis of AD, the generation of miRNA panel should be critical (Figure 2).

## 4. Alternative Splicing, Copy Number Variants (CNVs) in AD

Alternative splicing is important in the brain and may be influenced by aging and/or different environmental factors. Similar to DEGs, alternative splicing could play a role in brain functions, such as synaptic development and functions, or inflammatory pathways [62]. Gene expression alterations (either up- or downregulation) may also be involved in alternative splicing mechanisms by reduced DNA repair or by chromosomal instability [63]. Alternative splicing may result in the expression of different RNA/protein isoforms. Even though the majority of human genes are alternatively spliced, abnormal isoforms could be associated with disease onset [64]. Alternative splicing may occur through various mechanisms, including skipping the exons, alteration in acceptor-or donor sites, usage of mutually exclusive exons or intron retention [65]. In the brain, several AD-causing genes were verified to be affected by splicing and disease mechanisms, including *APP*, *PSEN1-2*, *APOE,* or *MAPT* [3,64]. Mutations/variants in *PSEN1*, located near the 5′ and 3′ may eliminate the acceptor or donor splicing site, and result in exon skipping. One of the most known splice site mutations in PSEN1 is the deletion of exon 9 (Ser290Cys) or exon 9 -10 (Ser290Trp), associated with aggressive AD phenotypes [66,67]. Additional exon-skipping mutations were also described, which could result in abnormal splicings, such as Leu113_I114insTyr [68], Leu271Val [69], or Ile416Thr [70]. *PSEN2* Lys115frameshift was verified to cause alternative splicing in the brain, which may result in reduced expression of wild type *PSEN2* protein. This mutation could result in the exclusion of *PSEN2* exon 6 [71]. The *MAPT* gene encodes the Tau protein has six different isoforms generated by alternative splicing of exon 2, 3 and 10. Splicing of exon 10 could generate the 3R and 4R, having 3 or 4 microtubule repeats, respectively. Balance of 3R and 4R is essential for the normal brain function, and changes in this ratio may result in alteration of APP dynamics. 3R and 4R Tau may enhance the anterograde and retrograde movement of APP, respectively. It may be possible that the Tau imbalance could impair the axonal transport of APP [72]. *MAPT* Asn279Lys mutation could alter the Tau exon 10 splicing, resulting in an imbalance in the 4R/3R-tau expression and neurodegeneration [73]. *ABCA7* is a strong risk factor for AD, where several mutations, including frameshift and nonsense variants, were observed. A variable number of tandem repeats (VNTR; 300 bp to 10 kb), located on intron 18, was suspected of playing a role in *ABCA7* pathogenicity. This VNTR could impact the expression of *ABCA7*, since its length may reduce the *ABCA7* expression. In addition, the longer VNTR may result in alternative splicing by affecting the cryptic donor site in intron 18, resulting in the partial or complete skipping of exon 19 [74]. A rare intronic variant in *ABCA7* (rs200538373) may be associated with abnormal splicing of exon 41. The heterozygous CG allele of this variant may result in an addition of 14bp to exon 41. Since the extra 14 bp also contained the STOP codon, it may result in a truncation of *ABCA7* [75]. *SORL1* may also be affected by alternative splicing. An intronic variant, c.1211+2T>G (located in intron 8), is located at the splice site of exon 8. The mutation could result in different effects: (1) full exon skipping, (2) the removal of 5 nucleotides in the 3′ end of exon, and (3) addition of 11 intronic nucleotides. These alterations may result in a nonsense codon and incomplete protein. Another variant c.3947-3insG is located in the splice site of exon 29, and the variant may result in the disruption of 3′ splice site and exon skipping. Missense variants may also affect the *SORL1* splicing, e.g., Asn1422Ser may generate a new 3′ splicing site [66]. Variants located in the splicing regulator elements of *EXOC3L4* may be cause exon skipping. This could result in reduced lysosomal transport and the removal of abnormal amyloid peptides [76].

Several variants and alternative splicing forms could result in a lower risk of neurodegeneration. For example, a variant on HMG-CoA reductase (*HMGCR*) enzyme gene rs3846662 could result in the alternative splicing and deletion of exon 13. The G allele of this variant was suggested to play a preventive role in disease onset in women [77]. *CD33* alternative splicing may also protective against AD. Two variants (rs3865444A and rs12459419T) were suggested to result in the splicing of *CD33* and modify the ratio of CD33 isoforms (full CD33 and truncated CD33). These SNPs favor the truncated form of CD33 or CD33m, which may result in elevated activation of microglia and enhanced amyloid clearance [78].

Copy number variants (CNVs) are deletions, duplications, or multiplications of a certain fragment of the human genome, and their size ranges between one kilobase to several megabases. They can involve one or several genes, but the majority of CNVs may affect the centromer or telomer regions. CNVs could play a key role in phenotypic diversity by altering gene organization and gene expression. In addition, CNVs may also increase the risk for different neurodegenerative disease phenotypes, such as ALS, autism, PD, or AD. Several known AD risk genes could be affected by CNVs, but genome-wide association studies have also discovered potential AD-related CNVs [79]. *APP* duplication may be associated with EOAD. Duplication of chromosome 21 was suggested to increase amyloid deposition at early ages in Down syndrome patients. However, the exact mechanisms of early onset dementia and amyloid deposition may also be associated to independent factors from *APP* trisomy [80]. Additional AD risk factors may also carry CNVs, which could be involved in AD onset. A low copy repeat associated CNV in complement receptor 1 (*CR1*), resulting in the CR-1S isoform, could also impact the AD risk. This isoform could be associated with increased C3/C4 binding sites and, possibly, reduced neuroprotective effects [81]. *CHRNA7* (neuronal nicotinic cholinergic receptor 7) is involved in schizophrenia and epilepsy, and it carries a duplication that may also be involved in AD onset. This gene may play a role in amyloid accumulation [82]. Swaminathan et al. analyzed CNVs on brain samples from late onset AD (LOAD_ and mild cognitive impairment (MCI) patients. GWAS studies revealed that potential candidate CNVs were found in AD patients, such as CHRNA7 And FAM7A Fusion (*CHRFAM7A*), DOP1 Leucine Zipper Like Protein B (*DOPEY2*), Reelin (*RELN*), Major Histocompatibility Complex, Class II, DR Alpha (*HLA-DRA*) [83]. Ghani et al. (2012) found an interesting association between AD and a duplication located on chromosome 15q11.2. This region contained five genes: Tubulin Gamma Complex Associated Protein 5 (*TUBGCP5*), Cytoplasmic FMR1 Interacting Protein 1 (*CYFIP1*), NIPA Magnesium Transporter 1-2 (*NIPA2* and *NIPA1*), and WAS Protein Homolog Associated With Actin, Golgi Membranes And Microtubules Pseudogene 3 (*WHAMML1_*, and gene expression analysis by quantitative PCR revealed elevated dosage for *CYFIP1* and *NIPA1* genes [84].

## 5. Methods That Could Be Used in Transcriptome Studies

Gene expression profiling of blood and brain tissue samples may help to find out the genetic–environmental relationship. Differently expressed genes and non-coding RNAs were suggested to play a role in disease pathogenesis. Prior to analyses, RNA should be extracted from tissue. Techniques require different essential steps, including RNAase removal, disruption of RNA-DNA or RNA protein complexes, and DNA removal by DNase. Messenger RNA may be enriched from total RNA by polyA probes and rRNA depletion [74]. Different samples of tissues have been used in AD research, including blood, plasma, cerebrospinal fluid (CSF), serum, or brain cells (Table 2). Brain tissues are the most efficient markers, which strongly reflect the disease-associated transcriptome changes [85]. However, brain tissues can be collected postmortem only. Samples from peripheral tissues (e.g., blood, plasma serum) can be easily obtained from patients, and their role in transcriptome studies should also be investigated. However, the majority of brain proteins may not be expressed in peripheral tissues, which could also result in confusion in transcriptome data [86]. The stability of RNAs may depend on the postmortem interval (PMI, the time between death and biopsy). Long PMIs may affect the quality of samples and inaccuracies in brain transcriptome studies [87]. Preserving the RNAs is essential for array and sequencing technologies. Several approaches may be available for RNA stabilization, including formalin fixation, flash freezing, specific chemicals for preservation, and commercial compounds. Formalin fixation keeps the structure of cells by the cross-linking of DNAs/RNAs and proteins. However, it may not be an appropriate method to extract good quality RNA. Flash-freezing by liquid nitrogen or dry ice may be a useful method in good quality RNA preservation, but it requires a special freezing system and centralized sample gathering. In addition, RNA may be sensitive to brief melting, so homogenization should be performed at low temperatures. Chemicals, such as sulfate solutions or TRIzol, may be useful in removing RNAases from tissues and could result in good quality RNAs. The disadvantage of chemicals is that they may damage the tissue structure, and make the histomorphological analyses difficult. Commercial compounds could also be useful in preventing RNA degradation, such as RNAlater (Ambion), Allprotect tissue reagent (Qiagen), or the PAXgene (both for tissues and blood) system. These compounds could provide long-term storage of RNAs in tissues, especially at low temperatures, protect the RNA against RNases, and also facilitate the histopathological studies [27,88]. 

Several methods were developed to analyze the gene expression, such as real-time PCR, microarray screening, and RNA sequencing techniques (Table 3). Array technologies have been widely used in genetic analysis, not only in genotyping but also in linkage analysis or gene expression. Fluorescent-labeled oligonucleotide probes were designed to analyze large sequence regions, and they could be a rapid and simple method. These probes should be attached to a solid surface, such as glass or plastic slides [95,96]. The degree of expression could be counted from the intensity of fluorescent signals after the probe–sample hybridization. The advantage of array technology is that arrays require less labor intensity, and the sample preparation and data screening may be much simpler compared to sequencing methods. The disadvantage of arrays is that they require a reference transcript of an annotated genome sequence or expressed sequence tag library. In addition, arrays can only detect SNPs, DEGs, splice variants, or miRNAs, for which the probes were designed for. Arrays are unable to detect additional or novel variations and potential candidates [96,97]. The Affymetrix GeneChip array has been widely used in gene expression analysis, where the gene-level expression may be examined. Exon arrays have also been designed, where probes are designed for individual exons, and may provide a more sensitive approach in gene expression measurement [98]. MiRNA profiling could also be possible by array techniques. Probes could be designed for the miRNA of interest. MiRNA arrays may be more complicated than mRNAs since miRNAs have a short size. MiRNA array analyses should require the ligation of RNA adapters and miRNAs, and amplification by RT-PCR and RNA polymerase transcription. Additional arrays that do not require amplification step may be available but use splinted ligation instead [99]. Arrays may also detect CNVs with similar genotyping process, compared to SNP genotyping. Illumina platforms, such as Illumina Human610-Quad, Illumina HumanHap 650Y, or Illumina Human Hap550K, have been used by ADNI, Caribbean Hispanic studies, and Duke University studies, respectively. The Affymetryx 6.0 array was used in the Translational Genomics Research Institute (TGEN) study. However, it may be difficult to study CNVs by arrays due to the potential large error rate, and their detection ability may be limited [100]. DEGs in AD brain tissues have been monitored by Patel et al. (2019). This study used Affymetrix and Illumina arrays and compared the transcriptomic pattern of different diseases, such as Huntington’s disease, bipolar disease, or schizophrenia. This study found several AD specific DEGs and may be significant in understanding AD-related mechanisms. However, the sample size was low and it may be hard to map all brain tissues, and the study was based on predefined probes/publicly available transcriptomic data [101]. Li et al. (2018) compared the data from brain and blood transcriptomes, proceeded by Affymetryx array platforms, and found several common pathways between them, such as mitochondrial- or oxidative-stress-associated pathways. However, this study may be validated in larger cohorts [102].

RNA sequencing (RNASeq) tools are next-generation sequencing (NGS) technologies, which were developed to screen the transcriptomes in terms of different diseases. RNASeq techniques could be used for profiling the gene expression, alternative splicing analysis, sequencing targeted RNA molecules, and also for single-cell sequencing [95]. Directly sequencing the RNA molecules may be possible, but the majority of techniques are based on DNA sequencing. Sample preparation may also be more complicated in RNASeq technologies compared to the arrays. For RNA-based sequencing methods, cDNA synthesis is required, which should depend on RNA sequence and structure. Mapping of miRNAs/mRNAs may also be challenging since they can be degraded quickly. Direct sequencing of RNA requires relatively simple sample preparation. By polyadenylation, short and long RNAs could be both observed, followed by only the fragmentation step. Targeted RNA sequencing needs longer steps of sample preparation and a higher amount of RNA or cDNA. These processes could also require several probes, arrays, and steps for target selection. The hybridization efficacy may also depend on the regions of interest [103]. 

Different RNA sequencing tools could be available, such as Roche 454, Illumina, Helicos (DNA polymerase based), or PacBio, SoLid (ligase based). The selection of platforms may depend on the purpose of the experiment. Helicos or PacBio could be useful in single-molecule detection, while Illumina or SoLid needs multiple copies (ensemble-based) of DNA/RNA. Single-molecule sequencing platforms may have a higher risk for error rate, compared to ensemble-based methods. The low error rate may be essential in the case of miRNa sequencing. Illumina and Solid could provide a higher depth of sequencing. In the case longer reads are needed, PacBio or Roche454 may be the more useful tool [118]. Recent approaches have developed nanopore sequencing, which could provide a longer sequencing length with higher accuracy [104]. Reverse transcription (cDNA synthesis) may be performed on RNAs. Prior to sequencing, RNA of interest may be enriched, which can be followed by RNA-or cDNA fragmentation by specific RNases or DNases, respectively. An adapter can be added to the fragments on 3′ and 5′ sites. The cDNA fragments can also be amplified before sequencing, and molecular labels should be used for analyzing the gene expression [105]. The benefits of RNASeq techniques are that they have a generally simple workflow. Several platforms may be available in case of sequencing, and bioinformatics methods can also facilitate the easy analysis [103,106]. RNASeq methods were designed to overcome the main limitations of array technologies. In arrays, hybridization may be problematic; cross-hybridization may occur in case of similar sequences, resulting in false-positive data, and the requirement for a priori knowledge on the sequences, and in addition, the qualification of lowly and highly expressed genes may be problematic. RNASeq could provide the discovery of novel DEGs, non-coding RNAs, or alternatively spliced genes, and higher throughput and more precise quantification of gene expression [119]. Annese et al. (2018) used Illumina platforms to analyze brain DEGs and miRNAs, and discovered the gene- and platform-regulation in late-onset AD patients. This study identified more than 2000 DEGs involved in several processes, such as the regulation of the neural system and vesicle trafficking. They also found novel miRNA clusters among LOAD patients. This study may provide a better understanding of the pathways involved in LOAD. The limitation of this study was that they could not provide insights on the early stages of neurodegeneration [14]. A study from van Rooij et al. (2019) focused on the DEGs among hippocampal mRNAs from AD patients and controls by Illumina HiSeq2000 at 2 × 50 bp. They performed integrative network analysis based on gene-annotated modules, but this study may also need to be validated [24]. 

Several challenges may appear in RNASeq techniques, such as issues with repetitive elements in the sequences or alternatively spliced genes. Data processing and dealing with hardware and bioinformatics tools may be challenging [103]. Issues with the majority of brain transcriptome methods are that they do not reveal the heterogenic expression pattern among cells from different tissues and do not focus on cell-to-cell variability. Single-cell transcriptome sequencing could be used to discover transcriptional diversity of the human brain by profiling the gene expression in different individual cells [120]. Mathys et al. (2019) analyzed postmortem brain samples with both high amyloid deposition and with low or without amyloid pathology. This study performed the sequencing by droplet-based single nucleus RNA sequencing (scRNA-seq). Single-cell RNA expression analysis can provide a more detailed image of AD-related expression changes, and on the complexity of gene interactions. However, further studies may be needed on scRNA-seq since distinguishing the protective and pathogenic pathways may be challenging [25]. ScRNA-seq may provide higher resolution of expression pattern at single-cell level, but disadvantages could be the more complex and noisy data. Further bioinformatics tools are needed to analyze the scRNA-seq data, and make sure of its accuracy [120]. 

Even though the accuracy of RNASeq and microarray analyses is relatively high, validation of the data may be required by several publishers. Quantitative real-time PCR (qRT-PCR) could be useful in the verification of gene expression data. Consistency was observed between RNASeq and qRT-PCR or microarray data. However, the 3′ UTR region may be excluded from qRT-PCR analysis due to its high variability. RT-PCR may also be used on allelic expression or splicing analysis [107]. The qRT-PCR methods are relatively quick and sensitive analyses, but the quality of mRNA, the choice of reference genes may affect the data [108]. 

For miRNA detection, additional methods have been developed, based on nucleic acid amplification, such as rolling circle amplification (RCA), duplex-specific nuclease (DSN)-based amplification, loop-mediated isothermal amplification (LAMP), exponential amplification reaction (EXPAR), and strand-displacement amplification (SDA). These methods are isothermal amplification methods and can provide accurate real-time detection of miRNAs [109]. RCA uses unique DNA/RNA polymerase enzymes to form a long ssDNA/ssRNA with several tandem repeats to the primer of a circular template. This method does not need a thermal cycler or heat-stable polymerase. Branched RCA can use several primers and may be a more sensitive approach in the detection of highly similar miRNAs [110,111]. DSN signal amplification assay is based on the usage of special nucleases, which cleave double-stranded DNAs or RNAs. In these methods, miRNAs can hybridize with a fluorescently labeled DNA probe and form a heteroduplex, which can be cleaved by DSN enzymes. Then, miRNA would be released and may form a second heteroduplex, which would be cleaved. The hybridization–cleavage circles could result in isothermal signal amplification, and miRNAs could be detected quantitatively [112,113]. LAMP was suggested as a rapid and economical method for miRNA detection. LAMP contains a template ssDNA and sets of primers. In template RNA, there should be a complementary sequence of miRNA of interest. In the presence of miRNA and DNA polymerase, a strand displacement DNA synthesis and exponential amplification can proceed. This method uses 4–6 primers and 6–8 target sequences [114]. EXPAR, combined with quantum-dot-based sensors, could be a sensitive method for miRNA amplification, even at low concentrations. This method combines an extension by polymerase and single-strand nicking steps and provides 10^6^–10^9^-fold amplification in a short time [115]. EXPAR usually used SYBR Green for fluorescent detection, but it may have the disadvantage that it can increase the risk of nonspecific amplification. Quantum dots (QDs) can have a wide range of excitation, strong photochemical stability, and high quantum yield. QDs have the benefits of a lower noise signal, and the bound and free probes should not be separated [116]. SDA is a linear amplification technique, and its steps include nicking, polymerase extension, and strand displacement, and their repetitive actions [117]. Enzyme-free amplification methods, such as hybridization chain reaction (HCR) or catalytic hairpin assembly (CHA), have also been developed, where miRNA acts as the initiator of strand displacement and can open up the DNA hairpins by negative free energy and the formation of base pairs. Combining these amplification methods may improve the miRNA diagnosis since it can enhance the trace target molecule, provide a cheaper and faster detection method, and in addition, reduce the background noises [109].

Reference databases on gene expression are available online on NCBI, such as Gene Expression Omnibus (GEO, https://www.ncbi.nlm.nih.gov/geo/), Sequence Read Archive (SRA, https://www.ncbi.nlm.nih.gov/sra) on ArrayExpress (https://www.ebi.ac.uk/arrayexpress/). These databases contain data from different transcriptome studies, performed by array and RNASeq techniques. The majority of journals request the gene expression data deposition for reuse, which may produce new insights on the disease-associated biological mechanisms. Meta-analyses of the available datasets may provide more insights on DEGs, their involvement in different pathways, and their role in disease diagnosis and therapies [121]. Several bioinformatics tools were designed to analyze the alternations in gene expression, such as gene expression analysis (DGEA), gene-set analysis (GSA), R-tools, Geo2R, GeoDriver, or GSEA tools. Some of these tools are based on the statistically significant differences in gene expression, while several of them may not provide visual analyses [122]. 

## 6. Potential Impact of Transcriptomes in AD Therapies

Alternative gene expression may be a potential target for therapies. Modification of the expression of AD-associated could play a role in disease prevention and therapies. Several miRNAs could be beneficial effects on neurons, while others may be involved in neurodegeneration. Modulating the mRNA expression could be possible by miRNA stimulation or inhibition [38]. Normalization of tumor suppressor gene expression by miRNAs and inhibition of oncogene up-regulation has been used in cancer therapies. Stimulation may be done by synthetic miRNAs, while for repression, miRNA sponges, anti-miRNA peptides, or antisense mediated inhibitors should be used. Delivery of miRNA may be done by polymer-based, lipid, or viral carriers. In neurodegenerative diseases, the main issues are that synthetic miRNAs or miRNA inhibitor-associated therapies should cross the blood–brain barrier, they should resist the cellular nucleases, and their cellular uptake should be improved. Additionally, their toxicity should be reduced and abnormal immune reactions, non-specific bindings must be prevented [123]. There are several miRNA candidates for AD therapies, which were analyzed in cell or mouse models. The miR-455-3p was examined in neuroblastoma cells, and the authors suggested that it could play a role in neuroprotection by reducing the expression of mutant *APP* and the levels of both Aβ42 and Aβ40 peptides [124]. MiR-301 was injected with a lentiviral vector in the hippocampus of triple mutant mice (*APP* Swe *PSEN1* M146V, *MAPT* P301L). MiR-301 reduced the expression of *APP* and *BACE1* in the hippocampus and resulted in an improvement of cognitive functions and reduced anxiety in the mice. This miRNA also lowered the amyloid deposition in the hippocampus and subiculum of mice [125]. Treatment of MiR-132 on primary mouse–human neurons and Tau mutant neurons showed that miR-132 may be protective against amyloid toxicity, and could also reduce the Tau pathology. It could upregulate pathways associated with synaptogenesis, neuronal growth, and survival, and downregulate the pathways involved in neuronal death [52]. MiR-188-5p expression was downregulated in the brain of 5*FAD mice and AD patients. It was suggested that stimulating the expression of MIR-188-5p could restore the synaptic functions and may improve cognition [126]. These studies suggested that miRNAs could be useful in gene expression regulation, and may be potential treatment on AD by modifying the abnormally expressed genes. However, the majority of these miRNA candidates are in the experimental phase; none of them reached Phase 3 trials, according to the clinicaltrials.gov database [127]. Targeting microglia, including the TREM-APOE pathway, may be a promising approach in therapies against neurodegeneration. TREM2 could promote the microglia functions and regulate the immune functions. APOE could also be involved in amyloid clearance by increasing the phagocytosis of amyloid peptides. APOE could bind TREM2, resulting in enhanced amyloid phagocytosis. A deficiency of APOE or TREM2 could reduce the protective mechanisms. Stimulating the expression by MiR-155 of TREM2 and APOE could reduce the plaque deposits in AD mouse models [128,129]. 

## 7. Challenges and Future Insights

Transcriptomics analysis is essential in studying complex diseases, such as cancer or neurodegenerative disorders. In AD, besides the causative genes, several risk factors can contribute to disease progression. In addition, studying the additional factors, such as gene-expression alterations, non-coding RNAs, CNVs, or splicing, may also provide additional information on the disease-associated pathways. The issue with AD diagnosis is that the definite disease diagnosis can only be made after postmortem autopsy or biopsy of the brain. Several imaging, genetic, and proteomic markers have been suggested to improve the disease diagnosis during the lifetime. Such biomarkers could be the amyloid-positive PET, Tau-positive CSF, atrophy on MRI, or positivity for mutations in AD causing/risk genes. However, early disease diagnosis may be problematic; patients with dementia are usually diagnosed 2–3 years after the first symptoms appeared, and cases have remained undiagnosed/misdiagnosed. Further studies are needed on biomarkers, which could enhance accuracy AD diagnosis during the lifetime [86,130]. Studying transcriptomes may provide additional markers (DEGs, non-coding RNAs) that improve the diagnosis of AD. Transcriptomics could open a new avenue for the treatment strategies of neurodegenerative disorders, including AD [65]. Even though there may be some overlap, the transcriptomic pattern of individuals with normal aging is suggested to be different from those who are diagnosed with AD. In AD, several genes are downregulated, which encode metastable proteins, and could play a role in homeostasis, resulting in abnormal amyloid deposits, Tau phosphorylation, or inflammation. Finding out and modifying the pathways associated with gene expression should be important not only in disease diagnosis but also in therapies [131]. Several technologies, including arrays and sequencing, were developed in transcriptomics, which could provide accurate data on gene DEGs, alternative splicing, or miRNAs [95]. However, there may be limitations and challenges in studying transcriptomes. For example, the quality of RNA samples may be difficult to maintain, and transcriptome profiles from different tissues (blood vs. brain) could be variable. In addition, mRNA from blood may be unstable, and it could be difficult to get samples from human brain species [132]. Peripheral samples (peripheral blood, serum, plasma, or peripheral blood mononuclear cells) could be more accessible for searching AD biomarkers as well as mild cognitive impairment [130]. Studying the transcriptomic signature of blood may be a useful approach in for AD prediction and in early disease diagnosis [133]. Perturbation in peripheral transcriptome may be a promising marker for AD, for example, in genes involved in inflammation, cellular survival or apoptosis, and mitochondrial fission. However, transcriptome in peripheral region changes may not fully reflect the AD-related changes inside the brain, such as amyloid deposition, and several proteins may not be expressed in blood [134]. In addition, the treatment strategies associated with transcriptomics (miRNA stimulation or inhibition) are currently in the experimental phase; further studies may be needed on how gene expression modification could be effective in the human brain [123].

## Figures and Tables

**Table 1:** Examples on miRNAs involved in Alzheimer’s disease (AD).

Mechanism	miRNA	Possible Effect	Reference
Amyloid metabolism	miR106a and b, mir20 family, miR153	Negative regulators of APP expression, their overexpression reduced in decrease of APP levels	[40,41,43]
	miR-29 family, miR107, mir29	Negative correlation with BACE1 expression, they may be down-regulated in case of high BACE expression, resulting in Ab accumulation	[43,45,46]
	miR-7, miR-9, miR-34a, miR-125b, miR-146a, and miR-155	They may be activated by Ab peptides and induce the amyloid clearance	[47]
	miR-455-3p	Protective against amyloid –associated toxicity, maintains mitochondrial stability	[48]
Inflammation	miR-146	Promoting amyloidogenesis through inflammation	[49]
	miR-9	Anti-inflammatory miRNA, its downregulation may result in neuroinflammation	[50]
	MiR-101	Modifier of microglia in CNS	[51]
	miR-125b	Its upregulation could repress the TREM2 expression and induce chronic inflammation	[49]
Tau processing	miR-125b	Overexpression of miR-215b may induce Tau hyperphosphorylation and apoptosis by CDK5	[35]
	miR-132/212	Result in imbalance in Tau phosphorylation through NOS1 pathway	[52]
	miR-138	Was upregulated in AD patients, may promote Tau phosphorylation	[53]
	miR-922	Regulate the AD pathway through Ubiquitin C-Terminal Hydrolase L1 (UCHL1). Tau phosphorylation may correlate negatively UCHL1	[54]
	miR-146a	Dysregulation of miR-146a could be involved in Tau phosphorylation through Rho Associated Coiled-Coil Containing Protein Kinase 1 (ROCK1) (inhibition of ROCK1)	[55]
	miR-106b	MiR106b overexpression may inhibit amyloid associated Tau	[56]
	miR-128	Controls the BAG2 cochaperone, and the degradation of insoluble/ phosphorylated Tau	[57]
	miR-219	Binds to Tau and represses its synthesis. Reduction of MiR-219 is associated with enhanced Tau toxicity	[58]
Apoptosis	miR-193a-3p	Downregulated in AD, inhibits cell apoptosis/ toxicity	[59]
	miR-15	Mediator of apoptosis, but is downregulated in AD, represses BACE1	[60]
	miR-34a	Controls neuronal cell cycle through blocking Cyclin D1. Reduced in AD	[61]
	miR-377	Downregulated in AD. Promotes cell proliferation, inhibits apoptosis	[30]

**Table 2:** Sample types used in AD-transcriptome studies.

Sample Type	Benefits	Limitations	References
Brain tissue	Can be used in many approaches Single-cell sequencing can also be possible to reflect the brain changes Reflect clearly the AD-related changes in the human brain Able to detect DEGs, miRNAs, alternative splicing	Samples can be collected only in postmortem status PMI may be important in sample collection/quality Difficult to obtain samples	[85]
Blood	Easy to obtain Potential candidate in transcriptomic markers the prediction of early disease progression Able to detect DEGs, miRNAs, alternative splicing	Does not reflect clearly the brain-related changes Difficult to replicate	[86]
Plasma or plasma- extracellular vesicles	Easily accessible RNase protected environment May contain brain-specific biomarkers, especially miRNAs	Further studies may be needed in their role May not be useful to analyze mRNA	[89]
Serum	Easily accessible Possible source of AD miRNA biomarkers	May not be useful to analyze mRNA Lack of extensive studies	[90]
Peripheral blood mononuclear cells	Easily accessible Promising sources of AD-related biomarkers Useful for both mRNA and miRNA	May not reflect clearly the AD-related changes Further studies may be needed on their role in AD	[91,92]
CSF	Source of miRNAs Sensitive marker for brain miRNAs Can be obtained from living donors	May not be useful to analyze mRNA Harder to obtain than blood/plasma/serum samples	[93,94]

**Table 3:** Techniques used in transcriptomic analyses.

Techniques	Applications	Examples	Benefits	Limitations	Reference
Array techniques	DEGs Splicing miRNA analysis CNV	Affymetrix GeneChip Illumina Human610-Quad	Less labor-intensive Rapid and accurate technique	Limited to the probes Does not detect novel variants/DEGs Risk of cross-hybridization	[95,96,97,98,99,100]
RNASeq	DEGs Splicing miRNA analysis CNV	Roche 454, Illumina, Helicos, PacBio, SoLid, nanopore sequencing	Larger scale of variant detection Simple workflow of sequencing	Needs special sample preparation, data analysis Potential high error rate	[103,104,105,106]
qRT-PCR	DEG miRNA	Applied BioSystems BioRAD	Less labor-intensive Quick and sensitive method, can verify array and RNASeq data	Limited to the gene of interest	[107,108]
Isothermal amplification	miRNA	RCA, LAMP, EXPAR, SDA, CHA	Rapid and accurate miRNA detection	Limited to certain miRNAs	[109,110,111,112,113,114,115,116,117]
